# Knowledge and attitudes of lower Michigan primary care physicians towards dietary interventions: A cross-sectional survey

**DOI:** 10.1016/j.pmedr.2022.101793

**Published:** 2022-04-13

**Authors:** Megan R. McLeod, Lisa Chionis, Brigid Gregg, Roma Gianchandani, Julia A. Wolfson

**Affiliations:** a UCLA Department of Internal Medicine, UCLA Health/David Geffen School of Medicine, Los Angeles, CA, USA; b University of Michigan Medical School, Ann Arbor, MI, USA; c University of Michigan, Department of Pediatrics, Ann Arbor, MI, USA; d University of Michigan, Department of Internal Medicine, Ann Arbor, MI, USA; e University of Michigan School of Public Health, Department of Health Management and Policy, Ann Arbor, MI, USA; f University of Michigan School of Public Health, Department of Nutritional Sciences, Ann Arbor, MI, USA; g Johns Hopkins Bloomberg School of Public Health, Department of International Health, Baltimore, MD USA

**Keywords:** RDN, Registered Dietitian Nutritionist, DASH, Dietary Approach to Stop Hypertension, ANOVA, Analysis of Variance, USDA, United States Department of agriculture, BMI, Body Mass Index, IPE, Interprofessional Education, Dietary intervention, Nutrition, Medical education, Knowledge, Attitudes, Primary care

## Abstract

**Background:**

Dietary interventions are first-line therapies for the prevention and management of many chronic diseases, yet primary care physicians prescribe these interventions infrequently.

**Objectives:**

This study investigates primary care physicians’ current knowledge and opinions regarding the delivery of dietary interventions. This work aims to identify modifiable barriers to prescribing dietary interventions to prevent and treat diet-related diseases.

**Methods:**

We designed and fielded an anonymous, cross-sectional survey of faculty and resident physicians across the Internal Medicine, Family Medicine, and Pediatrics departments in three academic and community hospitals in lower Michigan. Data were collected between January 15 and April 15, 2019. Self-rated knowledge and attitudes were measured on a 5-point Likert scale. Objective scores were calculated for each question as percentage answered correctly among all respondents. Objective knowledge scores were compared based on participants’ years in practice.

**Results:**

Response rate was 23% (356 responses). The sample was 62.3% female and 75.3% non-Hispanic White, and 56.7% were age 40 or younger. Average objective knowledge score was 70.3% (±17.2) correct. Mean self-rated knowledge score was 2.51 (±0.96) on a scale of 1(Poor) − 5(Excellent). Overall agreement with a statement of importance of dietary interventions was 3.99 (±0.40) on a scale of 1 (strongly disagree) to 5 (strongly agree). A majority (91.7%) of respondents indicated they would like more opportunities to learn about the evidence supporting dietary interventions.

**Conclusions:**

Physicians desire to incorporate dietary interventions into their practice. Findings encourage the development of educational strategies to support dietary intervention use among primary care physicians.

## Highlights

• Physicians rarely counsel patients about dietary interventions despite the proven efficacy of such interventions.
• Physicians both over- and under-estimated their knowledge on different dietary interventions.
• >90% of participating physicians expressed interest in continuing education on dietary interventions.

## 1 Introduction

Suboptimal diet is the leading preventable risk factor for non-communicable diseases (Afshin et al., 2019), and greater emphasis on the use of dietary interventions in primary care may improve population health and help reduce healthcare expenditures. However, primary care physicians seldom prescribe or counsel patients about dietary interventions, and their rate of use has declined in recent years despite static pharmacotherapy use (Jackowski et al., 2018).

One barrier to employing dietary interventions is the scarcity of nutrition education during medical school (Adams et al., 2015). Instruction on diet and nutrition in US medical schools routinely fails to meet the National Academy of Sciences’ recommended 25-hour requirement (Adams et al., 2015, Crowley et al., 2019). However, lack of medical education is only one factor contributing to the downward trend in dietary intervention use in primary care. Several studies attempting to understand this trend have cited systems-level barriers including concerns about visit length, billing, and lack of access to allied health professionals (Kushner, 1995, Schoendorfer et al., 2017).

Efforts to increase the use of dietary interventions by primary care physicians will require a more robust understanding of physicians’ current knowledge, opinions, and perceived barriers to their use (Levine et al., 1993). Several studies have surveyed physicians (cardiologists and internists) to assess objective knowledge and subjective attitudes towards dietary interventions (Harkin et al., 2018, Devries et al., 2017). The current study employs a large regional survey to investigate physicians’ knowledge, personal views, and perceived physician-level and systems-level barriers to employing dietary interventions in the context of primary care. Study aims include exploring demographic and general practice area-specific factors that may influence knowledge and attitudes towards dietary interventions and identifying potentially modifiable barriers to utilizing nutritional counseling as both a means of preventive care and disease management. Findings will contribute to the knowledge base on the application of dietary and lifestyle interventions in medicine and may inform future decisions in medical education and health policy.

## 2 Methods

### 2.1 Survey design

We designed a 50-item (<10 min) survey containing five sections: 1. Baseline information regarding each physician’s current practice 2. Self-perceived knowledge 3. Attitudes 4. Knowledge base 5. Demographics. The survey was tested for accuracy, face validity, and appropriateness by four physician experts, and was further tested and refined based on cognitive interviews among four fourth-year medical students at the University of Michigan.

### 2.2 Survey distribution

The survey was administered online via Qualtrics to residents and faculty in the departments of Internal Medicine, Pediatrics, and Family Medicine at Michigan Medicine, Family Medicine at St. Joseph Mercy Ann Arbor (MI), and Internal Medicine and Family Medicine at Western Michigan Medical School in Kalamazoo, MI. We contacted internal and family medicine department chairs at each institution via e-mail, and with their permission, introduced the survey to faculty and trainees via in-person meeting or via e-mail according to each chair’s recommendation. In total, 1,577 practicing physicians received the survey and 356 surveys were submitted between January and April of 2019, for a response rate of 23.14%. Incomplete surveys were excluded from this analysis (56). All respondents reported seeing at least one patient per day who was diagnosed with obesity, hypertension, diabetes, irritable bowel syndrome, or food insecurity. This study received exempt status from the University of Michigan Institutional Review Board (HUM00154960).

### 2.3 Measures

The full survey instrument is available in the supplemental materials. Self-rated knowledge scores were based on a 0–5 point Likert scale, where 0 indicates that the respondent is not aware of when the dietary intervention is indicated, 1 = poor knowledge, and 5 = excellent knowledge. Objective knowledge score was calculated using four true/false questions which were felt to be fundamental nutrition knowledge by the expert panel. Dietary intervention questions included in the self-rated and objective knowledge sections were similar but not identical. Attitudes were measured using a 5-point Likert scale, with 1 = strongly disagree and 5 = strongly agree. A 5-point Likert scale was additionally used to assess the following: ability to counsel low-income patients on specific resources for addressing food insecurity (Supplemental Nutrition Assistance Program (SNAP), Double Up Food Bucks (DUFB), and Prescription for Health 2022), self-rated proficiency in dietary counseling, satisfaction with the amount of dietary counseling employed in their practice, and referral rates to registered dietitian nutritionists (RDNs).

### 2.4 Statistical analysis

Mean objective knowledge scores were compared based on physicians’ years in practice using Analysis of Variance (ANOVA), and 95% confidence intervals were calculated based on this analysis. Physician attitudes were re-scaled from five categories to three categories, where higher numbers indicated an increased level of agreement with statements and general positivity towards dietary interventions. Differences in attitudes scores were examined based on demographic characteristics, and objective and subjective knowledge scores using ANOVA. All analyses were performed using R version 3.5.0 (R Foundation for Statistical Computing, Vienna, Austria).

## 3 Results

The characteristics of the study sample and referral rates to RDNs of the 300 respondents in the final analytic sample are presented in Table 1. The sample was 62.3% female, 75.3% non-Hispanic White, 67.7% physicians who primarily treat adult patients, 56.7% physicians with fewer than ten years of experience, and 35.5% physicians who saw <10 patients per week in the ambulatory setting. The composition of the sample based on physician specialty was 43.7% Internal Medicine, 22.3% Family Medicine, and 34.0% Pediatrics. Over half (56.7%) of participants were age 40 or younger. Many respondents (62.7%) report relying on a dietitian to provide patients with nutritional counseling, yet 54.7% report referring patients to a dietitian at less than half of visits.

**Table 1:** Characteristics of the study sample and referral rates to RDNs. (n = 300)*.

	Overall		Internal Med.		Family Med.		Pediatrics	
	N	%	N	%	N	%	N	%
TOTAL	300	100	131	43.7	67	22.3	102	34.0
Age (%)								
20–30	69	23.0	32	24.4	9	13.4	28	27.5
30–40	101	33.7	46	35.1	23	34.3	32	31.4
40–50	55	18.3	2	16.0	18	26.9	16	15.7
50–60	38	12.7	17	13.0	6	9.0	15	14.7
60–70	24	8.0	10	7.6	9	13.4	5	4.9
>70	5	1.7	2	1.5	0	0	3	2.9

Gender (%)								
Female	187	62.3	65	49.6	44	65.7	78	76.5
Male	104	34.7	63	48.1	21	31.3	20	19.6

Race (%)								
Asian	42	14.0	25	19.1	4	6.0	13	12.7
Hispanic	13	4.3	5	3.8	4	6.0	4	3.9
Black	5	1.7	1	0.8	3	4.5	1	1.0
White	226	75.3	94	71.8	52	77.6	80	78.4
Prefer to self-describe	2	0.7	2	2.5	2	3.0	0	0

Years in practice (%)								
<10	170	56.7	80	61.1	28	41.8	62	60.8
10–20	59	19.7	25	19.1	21	31.3	13	12.7
20–30	37	12.3	13	9.9	8	11.9	16	15.7
>30	7*	2.4	3	2.3	2	3.0	2	2.0

Majority of patient population (%)								
Adult	203	67.7	127	96.9	63	94.0	13	12.7
Pediatric	91	30.3	2	1.5	1	1.5	88	96.3

Patients seen per week in the ambulatory setting (%)								
<10	106	35.5	59	45.0	2	3.0	45	44.1
10–25	71	23.7	29	22.1	14	20.9	28	27.5
26–55	70	23.3	28	21.4	25	37.3	17	16.7
>55	43	14.3	11	8.4	24	35.8	8	7.8

How often do you refer patients to Dietitians								
Never	2	0.7	2	1.5	0	0	0	0
Sometimes	100	33.3	39	29.8	29	43.3	32	31.4
About half the time	62	20.7	31	23.7	12	17.9	19	18.6
Most of the time	98	32.7	40	30.5	21	31.3	37	36.3
Always	30	10.0	16	12.2	3	4.5	11	10.8

*Those who responded “prefer not to say” or “NA” for each measure were excluded from analysis.

Table 2 compares self-perceived and objective knowledge scores for various dietary interventions. P-values represent comparison of objective knowledge score based on years in practice as a physician (ANOVA). Overall self-perceived knowledge score was 2.50 ± 0.96, and average objective knowledge score was 70.3%. There was a significant difference in knowledge of the keto diet (p = 0.01) between physicians based on years in practice but no other observable differences in individual knowledge based on years in practice (Table 2). Self-perceived knowledge scores were lowest for the food insecurity questions, where average score was 1.5 for SNAP, 1.1 for Double-up food bucks, and 1.1 for Prescription for Health knowledge (Prescription for Health 2022), placing their self-rated ability to address patients’ food insecurity as between “Poor” and “Fair”.

**Table 2:** Self-perceived proficiency and objective knowledge scores for specific dietary interventions and topics based on years in practice as a physician. (n = 300)*.

Years in practice	Overall	<10	10–20	20–30	>30	*p*-value^Δ^
	N = 300* (%)	n = 170 (%)	N = 59 (%)	N = 37 (%)	N = 27 (%)	
**Mean Knowledge scores**						
Objective Knowledge % correct (95% CI)	70.3	68.3 (65.6–71.0)	73.3 (68.8–77.9)	73.7 (67.9–79.6)	72.0 (64.8–79.1)	0.145
Self-rated knowledge score	2.50	2.34	2.63	2.85	2.88	

**Perception Questions**						
Overall Positivity Score** (scale 1–5)	3.99	3.99 (3.93–4.06)	3.99 (3.88–4.10)	3.92 (3.78–4.06)	4.01 (3.84–4.18)	

Individual knowledge questions*						
**DASH**						
Don’t know (%)	5.7	4.7	6.8	13.5	0	
Poor (%)	18.3	23.5	13.6	8.1	14.8	
Fair (%)	11.3	14.1	13.6	0	7.4	
Neutral (%)	16.7	18.2	13.6	18.9	14.8	
Good (%)	32	31.8	37.3	37.8	22.2	
Excellent (%)	8.3	4.7	11.9	10.8	22.2	
**Objective score %**^†^ **(95% CI)**)	95.7	95.8 (92.5–98.9)	94.7 (89.1–100.0)	100.0 (92.9–107.1)	90.9 (82.3–98.8)	0.424

**Portion control**						
Don’t know (%)	1.3	0.6	5.1	0	0	
Poor (%)	7.3	10.0	5.1	2.7	3.7	
Fair (%)	4.3	15.9	3.4	8.1	11.1	
Neutral (%)	13.0	14.7	15.3	8.1	7.4	
Good (%)	43.3	40.6	49.2	51.4	48.1	
Excellent (%)	15.7	15.3	18.6	18.9	11.1	
**Objective score %**^‡^ **(95% CI)**	32.1	32.7 (25.0–39.6)	33.3 (22.1–47.0)	33.3 (17.2–49.4)	21.7 (3.02–41.4)	0.809

**Macronutrients**						
Don’t know (%)	4.6	3.5	6.8	10.8	0	
Poor (%)	31.7	35.9	32.2	4.3	22.2	
Fair (%)	15.7	18.2	13.6	13.5	11.1	
Neutral (%)	15.7	15.9	18.6	13.5	14.8	
Good (%)	19.0	19.4	0.3	13.5	25.9	
Excellent (%)	5.7	4.1	5.1	13.5	7.4	
**Objective score %**^§^ **(95% CI)**	72.3	68.5 (61.4–75.3)	86.4 (64.5–88.2)	75.4 (60.4–91.1)	75.8 (67.6–105.1)	0.282

**Keto/Saturated fat**						
Don’t know (%)	7.3	7.1	0.2	8.1	3.7	
Poor (%)	47.0	59.4	37.3	27.0	29.6	
Fair (%)	16.7	14.7	22.0	18.9	18.5	
Neutral (%)	7.7	4.7	10.2	13.5	14.8	
Good (%)	9.0	7.6	15.3	8.1	7.4	
Excellent (%)	4.7	3.5	1.7	13.5	7.4	
**Objective score %**^¶^ **(95% CI)**	53.9	46.7 (38.3–53.6)	64.9 (52.3–78.5)	69.7 (52.8–86.6)	59.1 (38.4–79.8)	0.0137

*Those who responded “prefer not to say” or “NA” for each measure were excluded from ANOVA analyses.

**Self-perceived knowledge scores were based on a 0–5 Likert scale, where 0 indicates that the respondent is not aware of when the dietary intervention mentioned is indicated, 1 = poor knowledge about the intervention, 2 = fair, 3 = neutral, 4 = good and 5 = excellent knowledge. Objective knowledge score was calculated as the mean percentage answered correctly using the true/false questions (answers) listed below.

**Δ p-values represent comparison of objective knowledge score based on years in practice using ANOVA. 95% confidence intervals are listed below the mean objective knowledge score.**

†Combining antihypertensive medication with the DASH diet is better than either intervention alone at reducing hypertension. (T).

‡The USDA’s MyPlate program recommends that ⅔ of each plate consist of fruits and vegetables.^53^ (F).

§Protein is the most energy dense food (calories/gram). (F).

¶ Foods that contain unsaturated fat include red meat and dairy. (F).

A large majority (95.6%) of respondents correctly identified that the DASH diet combined with antihypertensive medication is better than either intervention alone at treating hypertension, though only 44% (n = 121) rated their own knowledge of this concept as “Good” or “Excellent” (Table 2). In contrast, only 32.1% knew the correct portion of vegetables recommended by the United States Department of Agriculture’s (USDA) MyPlate, though 64% (n = 177) rated their knowledge as “Good” or “Excellent” (Table 2). Attitudes towards the use of dietary interventions were generally positive, and there was no difference in the overall attitudes score for physicians based on gender, years of experience, or specialty (Table 2).

The majority (91.7%) of all respondents reported that that they would be interested in opportunities to learn more about the evidence supporting dietary interventions, 89.2% said they would take an opportunity to learn more about how to counsel their patients about dietary habits, and 86.2% said that they would take an opportunity to learn more about how to counsel food-insecure patients (data not shown).

## 4 Discussion

We used a cross-sectional survey of Family Medicine, Internal Medicine, and Pediatric physicians at three Southeast Michigan hospital systems to contextualize the limited use of dietary interventions among primary care providers and to add granularity to previous findings regarding the lack of nutrition knowledge among general practitioners, medical residents, and medical students (Conroy et al., 2004, Vetter et al., 2008). This study adds to the previous work among primary care providers to examine the knowledge and attitudes towards use of dietary interventions (Antognoli et al., 2017, Ball and Leveritt, 2015, Cassidy-Vu and Kirk, 2020, Devries et al., 2017, Smith et al., 2015, Tronieri et al., 2019).

Physicians of varying levels of experience did not accurately rate their knowledge about specific nutritional interventions. Given mounting evidence supporting the addition of nutrition curricula to undergraduate medical education, one may anticipate that younger physicians might demonstrate superior nutrition knowledge than their predecessors, however our data do not demonstrate this trend (Sandhya et al., 2020). Physicians’ misjudgment of knowledge on the DASH diet and USDA portion sizes adds to existing evidence that physicians are not adequately educated about fundamental nutrition concepts and lack confidence in their ability to discuss evidence-based dietary patterns with patients. The discrepancy between objective and self-perceived dietary knowledge among physicians we have identified in our study warrants further investigation in larger sample sizes.

The physicians surveyed considered dietary interventions important and most respondents would pursue continuing medical education focused on nutrition. Some institutions do provide continuing nutrition education and online educational materials are freely available to all practicing physicians (e.g. Nutrition in Medicine (Kohlmeier et al., 2014)), though their popularity among our study sample is unknown. The objective knowledge gaps demonstrated in our findings suggest that including nutrition education as a core requirement in undergraduate and graduate medical education may be essential to improving use of dietary interventions or at least increasing RDN referral rates (DiMaria-Ghalili et al., 2013, Frantz et al., 2015). Culinary medicine electives have proven effective at training medical students and physicians in dietary counseling techniques (Birkhead et al., 2014). Similarly, online tools for learning about nutrition have also been successful in achieving high levels of participation among medical trainees (Lewis et al., 2014).

Based on findings, the investigators launched a series of multi-faceted pilot initiatives to expand the nutrition education opportunities available to medical students and physicians at the home institution. The initiatives included the following: (1) an interprofessional seminar for first-year medical students on fundamentals of the Mediterranean diet, DASH diet, and nutritional counseling based on motivational interviewing, (2) a Culinary Medicine elective for third- and fourth-year medical students offering hands on kitchen sessions, Culinary Medicine didactics, and a large IPE session with RDN students, (3) a virtual Culinary Medicine elective for Internal Medicine residents, (4) a longitudinal, interactive nutrition curriculum for Pediatrics residents during the Covid-19 pandemic, and additional initiatives targeted at a broader audience outside of primary care. Our group plans to study the outcomes of each program and build upon those which demonstrate greatest benefit.

Strengths of the current study include our ability to survey providers across a broad spectrum of primary care specialties within both academic and community-based institutions. However, the generalizability of this study is limited by its sample size, single geographic region, and our response rate of <25%, though this is a high response rate compared to other physician surveys (VanGeest et al., 2007). It is also important to note that our sample has a slightly larger proportion of physicians who identify as being either White or female than are represented in the general physician workforce (AAMC, 2018).

Diet-related chronic diseases are among the leading causes of morbidity and mortality in the US. Dietary interventions are effective at improving clinical indicators of disease among relevant patient populations, even those administered during the time patients spend in the physician waiting room (Cohen et al., 2017). Our survey identified marked gaps in physicians’ knowledge about basic nutrition concepts and their inaccurate understanding of their own knowledge. Additionally, physicians surveyed estimated their ability to counsel food insecure patients as especially poor, a striking finding considering 10.5% of US households containing over 38 million Americans were food insecure in 2019 (USDA ERS, 2022). Fortunately, we found that physicians are interested in receiving more nutrition education. These findings underscore the need for more robust nutrition education during undergraduate and graduate medical education and highlight the need for additional action to address barriers limiting the incorporation of dietary interventions into medical care. These findings may guide institutions in their efforts to improve physician knowledge about diet.

## Funding

This work was supported by the National Center for Advancing Translational Sciences of the NIH [TL1TR002242].

## CRediT authorship contribution statement

**Megan R. McLeod:** Conceptualization, Data curation, Formal analysis, Funding acquisition, Methodology, Visualization, Writing – original draft, Writing – review & editing. **Lisa Chionis:** Writing – review & editing. **Brigid Gregg:** Conceptualization, Methodology, Writing – original draft, Writing – review & editing. **Roma Gianchandani:** Conceptualization, Methodology, Writing – original draft, Writing – review & editing. **Julia A. Wolfson:** Conceptualization, Methodology, Project administration, Software, Writing – original draft, Writing – review & editing.

## Declaration of Competing Interest

The authors declare that they have no known competing financial interests or personal relationships that could have appeared to influence the work reported in this paper.
