# Supplementary material for: Knowledge and attitudes of lower Michigan primary care physicians towards dietary interventions: A cross-sectional survey
Source: Prev Med Rep. 2022 Apr 13;27:101793. doi: 10.1016/j.pmedr.2022.101793 (PMC9152810; doi:10.1016/j.pmedr.2022.101793)
Supplement: Supplementary data 1 [file mmc1.docx]

**Supplemental Materials:**

**Supplemental Survey 1:** Survey instrument to assess knowledge and attitudes of primary care physicians towards dietary interventions

**Background:** Diet-related diseases such as obesity, hypertension, diabetes, and irritable bowel syndrome comprise a large portion of primary care visits in the United States. However, physicians seldom prescribe dietary interventions, and their rate of use has declined in recent years. No study has been conducted to assess physician knowledge and opinions pertaining to dietary intervention. Our study aims to identify key factors that influence physicians’ use of dietary interventions. Please note that we do not aim to endorse any specific diet.

**Data safety:** Your participation in this study is completely optional and anonymous. Your responses will be recorded and analyzed under a dummy ID and are only viewable to the study team for purposes of the study. By completing this survey, you agree to participate in this study. At any point you are free to stop participating. You can contact the Study PI for any questions (mrmcleod@umich.edu).

This survey contains four parts that together take about 10 minutes: 1. baseline information regarding your practice (~1 minute) 2. Self-perceived knowledge and proficiency with regard to dietary interventions (~2 minutes) 3. Attitudes pertaining to dietary interventions (~2 minutes) 4. knowledge base (~2 minutes) 5. demographics (~2 minutes). Thank you in advance for participating!

## Baseline use of nutrition interventions

1.1 Do you accept medicaid or medicare? Y/N/don’t know

1.2 Do you see at least one patient per day who has been diagnosed with obesity? Y/N/don’t know

1.3 Do you see at least one patient per day who has been diagnosed with hypertension? Y/N/don’t know

1.4 Do you see at least one patient per day who has been diagnosed with diabetes (type 2)? Y/N/don’t know

1.5 Do you see at least one patient per day who has been diagnosed with irritable bowel syndrome? Y/N/don’t know

1.6 Do you see at least one patient per day who is food insecure? Y/N/don’t know

1.7 When considering all of your outpatient visits, how often is the topic of diet introduced by either you or the patient (please report as a percentage)?

1. 0-25%
2. 25-50%
3. 50-75%
4. 75-100%

## 2. Self-perceived knowledge and proficiency with regard to dietary interventions

| 2.1  When it is indicated, how would you rate your ability to counsel patients on adopting the following diets? Please select don’t know if you are not sure when these diets are indicated. | Don’t know | Poor | Fair | Neutral | Good | Excellent |
| --- | --- | --- | --- | --- | --- | --- |
| 2.1.1 Mediterranean diet | 0 | 1 | 2 | 3 | 4 | 5 |
| 2.1.2 DASH (Dietary Approaches to Stop Hypertension) | 0 | 1 | 2 | 3 | 4 | 5 |
| 2.1.3 Portion control (USDA Healthy plate guidelines) | 0 | 1 | 2 | 3 | 4 | 5 |
| 2.1.4 Carbohydrate counting (T2DM) | 0 | 1 | 2 | 3 | 4 | 5 |

| 2.1.5 Ketogenic diet | 0 | 1 | 2 | 3 | 4 | 5 |
| --- | --- | --- | --- | --- | --- | --- |
| 2.1.6 FODMAPs (Fermentable Oligosaccharides, Disaccharides, Monosaccharides and Polyols) | 0 | 1 | 2 | 3 | 4 | 5 |
| 2.1.7 Elimination Diets | 0 | 1 | 2 | 3 | 4 | 5 |

| 2.2  When seeing low-income patients, how would you rate your ability to counsel patients on using the following resources? Please select don’t know if you are unsure what these resources are. | Don’t know | Poor | Fair | Neutral | Good | Excellent |
| --- | --- | --- | --- | --- | --- | --- |
| 2.2.1 SNAP (Supplemental Nutrition Assistance Program) | 0 | 1 | 2 | 3 | 4 | 5 |
| 2.2.2 Double up food bucks | 0 | 1 | 2 | 3 | 4 | 5 |
| 2.2.3 Prescription for Health | 0 | 1 | 2 | 3 | 4 | 5 |

| 2.3  Please rank your ability to discuss the practical aspects of the following: | Poor | Fair | Neutral | Good | Excellent |
| --- | --- | --- | --- | --- | --- |
| 2.3.1 Patients’ current eating habits | 1 | 2 | 3 | 4 | 5 |
| 2.3.2 Patients’ current cooking ability | 1 | 2 | 3 | 4 | 5 |

**3. Attitudes pertaining to dietary interventions**

| 3.1 Please rank your level of agreement with the following statements: | Strongly Disagree | Disagree | Neutral | Agree | Strongly  Agree |
| --- | --- | --- | --- | --- | --- |
| 3.1.1 Efforts that I make to counsel my patients on nutrition are effective | 1 | 2 | 3 | 4 | 5 |
| 3.1.2 It is important for physicians to learn about nutrition | 1 | 2 | 3 | 4 | 5 |
| 3.1.3 It is valuable for my patients to receive dietary information from me | 1 | 2 | 3 | 4 | 5 |
| 3.1.4 I rely on dieticians to provide guidance to my patients | 1 | 2 | 3 | 4 | 5 |
| 3.1.5 It is appropriate to counsel all patients about their diet | 1 | 2 | 3 | 4 | 5 |
| 3.1.6 I am more motivated to counsel an obese patient (BMI >= 30) about dietary interventions than a non-obese patient (BMI <30) | 1 | 2 | 3 | 4 | 5 |
| 3.1.7 Dietary interventions are important aspects of the care plan for many diseases | 1 | 2 | 3 | 4 | 5 |
| 3.1.8 I have time during outpatient visits to discuss my patients’ dietary habits | 1 | 2 | 3 | 4 | 5 |
| 3.1.9 I am satisfied with the amount of dietary counseling I do in my practice | 1 | 2 | 3 | 4 | 5 |

3.2 If you are unsatisfied (answered 1 or 2 in question 3.1.9) with the amount of nutrition counseling you use in your practice, what do you see are the most relevant barriers to employing those interventions in your practice? Please choose all that apply.

1. Time
2. Compensation
3. Self-perceived inefficacy
4. Knowledge deficits
5. Lack of comfort discussing diet
6. Other _______________

3.3 Please rank from 1-4 the following topics according to which you would prioritize in a yearly wellness visit (assume all are relevant to the patient’s care):

___ Smoking

___ Diet

___ Exercise

___ Vaccination

**4. Knowledge pertaining to Dietary interventions and guidelines**

**Please respond to the following as T/F:**

_____Combining antihypertensive medications with the DASH diet is better than either intervention alone at reducing hypertension.

_____Metformin is superior to lifestyle interventions in reducing the incidence of T2DM in pre-diabetic adults.

_____ Protein is the most energy dense food (calories/gram).

_____ Foods that contain unsaturated fat include red meat and dairy.

_____ The USDA’s MyPlate program recommends that ⅔ of each plate consist of fruits and vegetables.

_____ The USDA recommends that people consume <=2300 mg of salt daily.

**5. Demographics**

5.1.1 What is your age?

1. 20-30
2. 30-40
3. 40-50
4. 50-60
5. 60-70
6. >70
7. Prefer not to say

5.1.2 What is your gender?

1. Female
2. Male
3. Non-binary/third gender
4. Prefer to self-describe ________
5. Prefer not to say

5.1.3 Would you say you are:

1. Underweight
2. Healthy weight
3. Overweight
4. Obese
5. Prefer not to say

5.1.4 What is your race/ethnicity?

1. Non-Hispanic White
2. Non-Hispanic Black
3. Hispanic
4. Asian
5. Prefer to self-describe: ____________
6. Prefer not to say

5.1.5 How would you rate the quality of your own diet?

1. Poor
2. Fair
3. Average
4. Good
5. Excellent
6. Prefer not to say

5.1.6 Do you primarily work with adult or pediatric patients?

1. Adult
2. Pediatric

5.1.7 What is your current/intended specialty?

1. Primary care - adult
2. Family practice
3. Cardiology
4. Gastroenterology
5. Interventional cardiology
6. Hematology
7. Endocrinology
8. Rheumatology
9. Pulmonology
10. Nephrology
11. Infectious Diseases
12. Critical Care Medicine
13. Oncology
14. Geriatric Medicine
15. Allergy & Immunology
16. General Medicine
17. Undecided
18. Prefer not to say

5.1.8 How many years have you been in practice?

1. <10
2. 10-20
3. 20-30
4. 30-40
5. >40
6. Prefer not to say

5.1.9 Approximately how many patients do you see per week in an ambulatory setting?

1. <10
2. 10-25
3. 25-40
4. 40-55
5. 55-70
6. 70-85
7. >85
8. Prefer not to say

5.2.0 When it is indicated, how often do you refer your patients to dieticians?

1. Never
2. Sometimes
3. About half the time
4. Most of the time
5. Always
6. Don’t know/prefer not to say

5.2.1 If you had the opportunity to learn more about the evidence supporting dietary interventions, would you take it? *[yes/no]*

5.2.2 If you had the opportunity to learn more about how to counsel patients on their dietary habits, would you take it? *[yes/no]*

5.2.3 If you had the opportunity to learn more about how to counsel food insecure patients, would you take it? *[yes/no]*

5.2.4 Did you have experience in nutrition before becoming a physician? *[yes/no]*

*If yes to Q5.2.4*

In what area and how long was that experience? [open response]

Include your email below to be entered into a drawing for one of two digital $100 Amazon Gift cards. Emails will be recorded separately from survey responses. _________________
